# “One has to beg and fight for everything” – a qualitative study on patient journeys of children with chronic conditions in Germany

**DOI:** 10.1186/s12913-026-14386-5

**Published:** 2026-03-20

**Authors:** Carmen J. Herr, Freia De Bock, Angélique Herrler

**Affiliations:** 1 Department of General Pediatrics, Neonatology and Pediatric Cardiology, Medical Faculty and University Hospital Düsseldorf, Heinrich Heine University Düsseldorf, Moorenstraße 5, 40225 Düsseldorf, Germany; 2 Department of Pediatrics, University Medical Center of the Johannes Gutenberg University Mainz, Langenbeckstraße 1, 55131 Mainz, Germany; 3 Charité – Universitätsmedizin Berlin, corporate member of Freie Universität Berlin and Humboldt-Universität zu Berlin, Institute of Medical Sociology and Rehabilitation Science, Charitéplatz 1, 10117 Berlin, Germany

**Keywords:** Child health, Child health services, Patient journey, Chronic disease, Intersectoral collaboration, Integrated health care systems, Health services accessibility, Care pathways

## Abstract

**Background:**

The rising prevalence of chronic conditions in children presents a major challenge for overburdened care systems. Despite high per-patient healthcare costs, affected children report lower health related quality of life and social participation compared to healthy children. By investigating children’s patient journeys in Germany, this study explores experiences of children and their caregivers with navigating care systems and identifies common challenges that may serve as leverage points for system improvement.

**Methods:**

In a cross-indication, cross-system approach, semi-structured interviews were conducted with a purposive sample of children aged between 3 and 17 years (*n* = 17) and their caregivers (*n* = 22) between May 2023 and February 2024. The investigated time period ranged from the first onset of symptoms to the current point in the care trajectory. Diagnoses included were ADHD, autism, developmental disorders, cerebral palsy, bronchial asthma, and type 1 diabetes. Verbatim interview transcripts were analysed using thematic analysis to identify patterns and key themes across participant groups.

**Results:**

Several factors were identified that lead to a burden for children with chronic conditions and their caregivers: (1) A fragmented healthcare landscape characterised by limited interdisciplinary and interprofessional collaboration and a lack of continuity in care. (2) The insufficient integration of services across healthcare, social services, and the education system, which places the responsibility on parents to bridge information gaps and coordinate services. (3) Limited availability of services, resulting in long waiting times and impeding timely, needs-based care according to child developmental stage and (4) the absence of a family-centred perspective on healthcare. This results in disproportional burdening of families with lower socioeconomic status, impeding access to healthcare, education, psychosocial well-being and long-term life opportunities for the child.

**Conclusions:**

Coordinated, timely, and developmentally appropriate care across medical, educational, and social systems is vital for children with chronic conditions. Interventions should align with the WHO framework on integrated, people-centred health services, ensuring cross-system continuity and addressing both child and family needs.

**Trial registration:**

This study was pre-registered in the German Register for Clinical trials (DRKS00032139) on 07 November 2023.

**Supplementary Information:**

The online version contains supplementary material available at 10.1186/s12913-026-14386-5.

## Background

The increasing prevalence of chronic conditions in children [1] poses a challenge in the face of already overburdened healthcare systems, particularly due to cross-system care needs requiring coordination across medical, educational, and social services. Moreover, despite high costs per patient for healthcare providers, children with chronic conditions and their families often experience significant, potentially avoidable additional burden due to complex coordination efforts and reimbursement procedures [2–5]. Having a chronic somatic or mental health condition is often associated with a lower social participation [6] and can lead to a lower health-related quality of life for children [7]. Chronic conditions can negatively impact school performance and thus affect job prospects and increase socioeconomic inequalities [8]. Moreover, caring for a child with a chronic condition can place a burden on the whole family, leading to parental distress [9], significant out-of-pocket expenses and additional expenditure of time for care work [10]. Studies examining the healthcare spending for children have found costs for children with complex needs for medical care to comprise up to a third of healthcare disbursals for all children [11]. Hence, establishing a reliable and efficient healthcare infrastructure for children with chronic conditions is crucial to improving healthcare services and patient outcomes as well as cutting costs.

To investigate the course of healthcare utilization and explore how it is experienced by patients and families, the concept of “patient journeys” can be applied. Patient journeys, in the context of this study, are defined as “the spatio-temporal distribution of patients’ interactions with multiple care settings over time” [12], encompassing the entirety of interactions of a patient with agents of the care system from the occurrence of the first symptoms until the end of therapy, treatment or assistance.

To date, little is known about children’s patient journeys, yet such insights can be instrumental in guiding improvements in care. So far, most research on patient journeys has primarily focused on patients with a specific, oftentimes rare condition (e.g. limb-girdle muscular dystrophy [13], osteogenesis imperfecta [14], congenital diaphragmatic hernia [15] or glycogen storage disease [16]). These studies were mostly focusing on the journeys of patients within the inpatient healthcare sector. Given the rarity of these conditions, studies that exclusively examine paediatric patient journeys—and the specific challenges faced by children and their families—remain scarce [17]. Moreover, the studies differ with respect to in how far patients’ perspectives are included in the scientific investigation, dependent on which definition of the term “patient journey” is employed [18]. Most studies which include children are situated in high-income countries such as the US [19], Canada [13], UK [15, 16] or European countries [14, 17, 20], but data on children’s patient journeys situated in Germany is lacking. Studies on patient journeys exclusively situated in the German healthcare context have so far only examined adult patients [21–23].

Hence, there is a lack of knowledge on children’s patient journeys that hinders identification of problems for patients and families associated with the healthcare system. This impedes further enhancement of services for children which could improve patient outcomes and improve efficacy of services [24].

Therefore, this study aimed to investigate the patient journeys of children with chronic conditions, and the health, educational and social care landscape in which they are situated from a cross-indication, cross-system perspective. The research question guiding the study was: What are shared characteristics of the patient journeys of children with chronic conditions in Germany, particularly in relation to cross-system care and its impact on family life?

## Methods

### Study design and setting

This study employed an explorative qualitative research design to investigate healthcare experiences and processes from the perspectives of children and their caregivers. Semi-structured interviews with children with chronic conditions and their caregivers were conducted and analysed using thematic analysis according to Braun and Clarke [25].

This study was situated in Germany. The German health care system is structured in an outpatient, inpatient and rehabilitation sector. With a quota of nearly 90%, the majority of the population is statutorily insured [26]. Besides necessary medical treatment, statutory insurance covers expenses for medically based health prevention and promotion, dental treatment, medication, aids and home nursing care, according to a benefits catalogue defined by the Federal Joint Committee (Gemeinsamer Bundesausschuss) [27].

The study received ethical approval from the Medical Ethics Review Board of the Medical Faculty of Heinrich-Heine-University Düsseldorf (2022–2290). It was pre-registered in the German Register for Clinical trials (DRKS00032139). Written informed consent was obtained by all participants. For interviews with children, written informed consent was obtained both from child and caregivers. Reporting in this study follows COREQ criteria [28].

### Sampling & recruitment

Sampling followed a purposive sampling approach [29]. Special attention was devoted to diversity of the sample concerning sociodemographic characteristics (e.g., age) as well as type and severity of the diagnoses.

Eligibility was based on diagnosis of the child (including ADHD, autism, developmental disorders, cerebral palsy, bronchial asthma, and type 1 diabetes), age of the respective child between 3 and 17 years and the ability to participate in an interview in German language. While recruitment was open to this full age range, in practice only children aged 6–17 years took part. Younger children were included indirectly through their parents. The diagnostic groups were selected to represent chronic conditions commonly encountered in paediatric populations and to reflect a broad spectrum of healthcare needs, ranging from low-complexity care (e.g., speech therapy for a child with a speech disorder) to highly specialized, multidisciplinary care (e.g., severe, multiple conditions). This approach was taken to enhance transferability of findings to children with other chronic conditions, e.g., obesity. Eligibility criteria for children and caregiver are shown in Table 1.

**Table 1:** Eligibility criteria

Population	Inclusion criteria	Exclusion criteria
Child	- child aged 3–17 years with at least one of the following diagnoses: - behavioural and emotional disorders with onset usually occurring in childhood and adolescence (ICD-10: F90-94, e.g., ADHD) - bronchial asthma (ICD-10: J45) - diabetes mellitus type 1 (ICD-10: E11) - developmental disorders (ICD-10: F80-84, e.g., autism) - cerebral palsy (ICD-10: G80) - ability to participate in an interview of 15–30 min in German language	- none of the diagnoses included; - below 3 or above 17 years old; - lack of language skills or other problems (e.g., with concentration) to participate in an interview of 15–30 min in German language
Caregiver	- custody of a child aged 3–17 with at least one of the following diagnoses: - behavioural and emotional disorders with onset usually occurring in childhood and adolescence (ICD-10: F90-94, e.g., ADHD) - bronchial asthma (ICD-10: J45) - diabetes mellitus type 1 (ICD-10: E11) - developmental disorders (ICD-10: F80-84, e.g. autism) - cerebral palsy (ICD-10: G80) - ability to participate in a 30–60 min interview in German language	- no custody for the child in question; - no child in the above-mentioned age range with one of the above-mentioned diagnoses; - lack of language skills or other problems (e.g., with concentration) to participate in an interview of 30–60 min in German language

Abbreviations: ICD-10: International Statistical Classification of Diseases and Related Health Problems,10th revision; ADHD: Attention-Deficit/Hyperactivity Disorder

Sample size was approximated at 15 children and 15 parents based on empirical recommendations to achieve saturation [30, 31]. Children and caregivers were recruited through dissemination of leaflets and information on the study in social paediatric centres, outpatient departments of university hospitals and patient support groups across Germany.

### Design and development of the interview guide

For the development of the interview guide, a literature review was conducted and a preliminary set of interview questions was discussed in the multidisciplinary project consortium (consisting of experts from the fields of medicine, health sciences, law, health economics, health insurance, epidemiology). Eventually, three interview guides were derived: one for interviewing children alone, one for interviewing caregivers alone, and one for joint interviews, each with different foci. The interview guides were pretested with a child and a mother and revised regarding comprehensibility and scope. The pilot interviews were not included in analysis for reasons of comparability. Table 2 provides an overview of the structure and themes of the interview guides. The interview guides are provided in the appendix.

**Table 2:** Overview of structure and themes of the interview guides

Caregiver		Child and caregiver together			Child
*Clinical picture and diagnosis*					
Description of the condition of the child, clinical presentation					Description of the condition, clinical presentation
*Patient Journey*					
course of events from first symptoms to diagnosis and treatment, experiences with utilisation of care services and structures					symptoms, medical diagnosis, information given to the child on diagnosis and treatment plan, important figures providing support and guidance
*Everyday life*					
Particularities due to the condition of the child in everyday life, school or kindergarten, recourse to support (e.g. by domestic help)					
	Description of daily life, preferred activities, routines, limitations due to the condition, reconciliation of medical appointments and leisure activities				
*Social participation*					
Parental assessment of social participation of the child, barriers to social participation	Kindergarten/school life, child’s assessment of social participation, perceived barriers, suggestions for improvement, support needed to realise participation				
	Interaction with parents concerning the condition and therapy management, perceived autonomy				
*Healthcare experiences*					
	positive and negative experiences with the health care system and interactions with health care professionals, children’s involvement in decision making				
positive and negative experiences with the healthcare system, navigation of the healthcare system, organisation and coordination of care, involvement of parent and child in therapy and treatment decisions					
*Caregiver burden*					
Caregiver burden attributed to provision of healthcare: time and financial expenditure for care, personal health, reconciliation of work and care responsibilities, strain on siblings and other family members					
*Wishes*,* suggestions for improvement of care*,* priorities*					
Course of an ideal patient journey of a child with the same condition					Recommendations on how to improve care for a child in a similar situation?

### Data collection and analysis

Data was collected from May 2023 to February 2024. Potential participants contacted the study group and were invited for a short introductory meeting via telephone to assess eligibility for the study and set a date and location for the in-person interview (at their home or a facility where they receive healthcare). Both caregivers and children received a 25€ voucher as incentive. Interview format was determined by the families. When parents agreed to an interview with their child, the child was informed about the study and its purpose, including what participation would involve, and asked for their consent. In most cases, the child decided whether the interview would be conducted individually or together with a parent. The number of interviews conducted in each format can be found in Table 3.

All interviews were audio-recorded and transcribed verbatim. Interviews were conducted by CJH (physician, medical anthropologist) and AH (health scientist). There was no prior relationship to the participants. Analysis followed the concept of thematic analysis by Braun and Clarke [25, 32, 33]. Within the development of a thematic framework, we additionally employed the concept of thematic saturation [30]:

1. A base size of six randomly chosen interviews was independently coded by two researchers (AH, health scientist and CJH, physician, medical anthropologist; both female), inductively deriving semantic and latent codes from the dataset.
2. CJH and AH commonly sketched a thematic map [25] to determine the initial scope of information, identify shared codes and cluster codes into themes.
3. A new run with the length of three interviews, independently coded by AH and CJH, was stipulated to ascertain additional information. AH and CJH added all new codes to the thematic map and revised it accordingly.
4. We set the New Information Threshold (additional codes identified in a run length compared to the number of codes identified in base size) to ≤ 5%. Step 3 was repeated until the threshold was reached.
5. AH and CJH reviewed and reworked the themes clustered in the thematic map to create a codebook, defining and labelling all themes.
6. All interviews were coded by CJH supported by two further colleagues, using the final codebook. Coding was reviewed by AH.
7. CJH conducted an in-depth analysis of each theme and sub-theme across all interviews, comparing them by diagnosis group, to develop patterns and explain correlations between the described phenomena. This analysis was discussed in the research team and further refined by CJH.

Analysis of coded interview transcripts was guided by constructionist paradigm, aimed at identifying underlying structural conditions as well as socio-cultural concepts shaping the views and experiences expressed by participants [25]. Thematic saturation was monitored across participant groups, and recurring patterns emerged consistently despite the diversity. This suggests that the key themes identified reflect shared experiences across different diagnoses and ages, while also capturing condition-specific nuances. Heterogeneity of diagnosis groups, in particular “developmental disorders”, was taken into account during the analyses by examining autism spectrum disorders separately. However, as the primary aim of this study was to identify commonalities across indications rather than differences between specific diagnostic groups, results are presented using a unified "developmental disorders" category. In interviews involving children with multiple diagnoses, references were attributed to the specific condition being discussed, allowing condition-specific aspects to be considered within the broader cross-indication analysis. Pseudonymized interview transcripts were coded in MAXQDA^®^ version 22 (Verbi, Berlin). The results were discussed with members of a caregiver support group for children with rare conditions and a children’s council (group of children with chronic conditions and their parents). Participant quotes in this paper are translated to English. A table with the original German version can be found in appendix Table 1 [see appendix].

## Results

### Sample

Participants from different German federal states took part in this study. As interviews were conducted in person, the majority of participants was from North-Rhine-Westphalia, Germany’s most populous federal state. Other participants were from Rhineland-Palatinate, Hesse, Bavaria and Lower Saxony. In total, 22 parents (“P”, median age of their respective children 10 years, range 4–15 years), mostly female, and 17 children (“C”, median age 11 years, range 6–17 years), took part in the study. Details on the sample are represented in Table 3. The New Information Threshold was reached after 12 interviews. 16 additional interviews were conducted to enhance depth, richness, and representation across different chronic conditions [31, 34, 35]. Eight interviews did not solely cover one of the indications but concerned (a) several children of the family with different diagnoses or (b) a child with multiple (≥2) conditions included in this study.

**Table 3:** Sample characteristics

		Behavioural and emotional disorders (ICD-10: F90-94)	bronchial asthma (ICD-10: J45)	Diabetus mellitus type 1 (ICD-10: E11)	developmental disorders (ICD-10: F80-84)	cerebral palsy (ICD-10: G80)
*Number of interviews per interview mode*	total	5	6	4	12	9
	C	1	3	0	2	5
	C & P	3	2	3	3	2
	P	1	1	1	7	2
*sex of respective child*	male	3	5	2	6	8
	female	2	1	2	6	1
*age of respective child*	3–5	0	1	0	3	0
	6–10	4	0	2	6	4
	11–15	1	3	2	3	2
	16–17	0	2	0	0	3

### Macro-level conditions shaping patient journeys

One major theme concerned the macro-level structural and organisational conditions shaping access to and quality of care for children with chronic conditions. A central concern was the lack of coordination within and between systems, with families experiencing barriers not only in healthcare, but also across the social services and education system. Furthermore, participants pointed to insufficient service capacities and high rejection rates of applications and reimbursement claims impeding adequate and timely care. These structural conditions made parents feel like they had to fight against a hostile system which hindered rather than facilitated adequate care and support for their child appropriate for its developmental stage and requirements.

### Coordinating care within the medical system

Especially for children with complex or multiple chronic conditions (≥ 2 of the listed diagnoses), parents emphasized a lack of coordination and communication among disciplines involved in the treatment of their child. They criticized the absence of direct communication between allied health professionals and physicians, noting that parents were often relied upon to relay information. Parents remarked that this led to conflicting recommendations given by different disciplines – shifting responsibility for treatment decisions to families. Even more so, they felt health practitioners failed to look beyond the boundaries of their own specialisation to account for a *“holistic perspective”* (P5 + C3 § 163) on the needs of child and family.

In the perception of parents, the lack of interdisciplinary and interprofessional communication created a necessity for more in-person appointments with doctors and therapists. Hence, parents said they wished for more digital solutions in the healthcare system, e.g., video or phone appointments with doctors and an extended validity of letters of referrals (which to date have to be collected in person at the primary care provider every quarter).

In the account of parents, the organisation effort for them was especially high with regard to the provision of aids, e.g., wheelchairs or orthosis, which they can -in principle- be reimbursed for by their health insurance company. Yet, with doctors, assistive technology providers and health insurances involved, parents had to keep track of the status of processing of their request and check in with all the players involved to prevent a rejection of their claim due to missed deadlines.

Overall, parents unanimously said they felt responsibility for coordination and organisation of care rested on them alone. This impression was reinforced by the experience that care counselling offices, health and nursing care insurances often had insufficient knowledge on the needs of and existing services for children with chronic conditions.

### Coordinating care across the social services and education system

The coordination of care for parents involved managing not just medical needs but also navigating social services and the education system. Services administered in the social services system in Germany are, e.g., autism-specific therapy, day group care, socio-pedagogical family assistance or school assistants.

Families described that some of these services were difficult for them to reach. Many families mentioned difficulties navigating systems in the face of unclear responsibilities and inscrutable subject-matter jurisdiction. Even more so, parents felt confronted with an ever-changing thicket of regulations and responsibilities but not equipped with the tools to navigate it. As one parent put it: “*It’s like [the game] ‘musical chairs’”* (P4 + C2 § 759).

The allocation of responsibilities between systems, institutions, health and nursing care insurance seemed opaque to most parents interviewed, referring to it as *“totally incomprehensible”* (P6, § 73). Regarding authorisation of school assistance and day care access, caregivers reported that administrative responsibility shifted depending on the child’s diagnosis (e.g., from youth to social welfare office), which sometimes even led to exclusion from services when a child received an additional diagnosis.

Table 4 illustrates the distribution of responsibility for authorization of services for children with chronic conditions across systems based on the interview data.

**Table 4:** Responsibility for authorization of services across systems based on the interview data

healthcare system [36]	social services system	education system [37]
Health insurance: - medical treatment and therapy, - medical aids	Social welfare office [38]: - Disabled person’s pass - school assistant* - autism specific therapy* - day group care*	Disadvantage compensation in school
Nursing care insurance: - home nursing care products, - respite care, - prevention care	Youth welfare office [39, 40]: - school assistant* - autism specific therapy* - day group care*	Expert opinion on the determination of special educational needs
Medical service (MD): - allocation of care degree for nursing care insurance - upon request by health insurance: Examination of application for medical aids		

* Responsibility dependent on diagnosis and number of diagnoses of the child

### Systemic resource limitations

A topic thematised across interviews was a perceived lack of resources for children with chronic conditions. This applied to the medical as well as the social and education system.

With regard to medical care, parents critiqued long waiting times for diagnostics and treatment, especially for neuro-psychiatric conditions like autism and ADHD. Waiting time for a diagnostic appointment for autism was reported as one year or longer. Waiting for a therapy spot could take up to five years—by which point, as one parent remarked, their child *“would almost be an adult”* (P3 § 33).

Children also reported long waiting times for approval of medical aids by health insurance companies after their parents submitted cost-assumption requests. Parents recounted that in many cases, insurances called in the medical service (Medizinischer Dienst) for an examination of the request, resulting in a rejection of the claim. Appealing against the rejection was described by parents as time-consuming and potentially costly (in case they had to get a lawyer or go to court). Several caregivers reported that approval delays for replacing outgrown medical aids were so long that the new aids were already too small upon arrival.

The delay in diagnosis and therapy made parents fear that important development windows were passing without adequate support for the child. In some cases, caregivers reported that the lack of timely care even led to a regression of progress achieved in therapy, *“simply because the system doesn’t provide the help at the point where you need it”* (C8, § 252). They emphasized the need for quicker and timely approval processes, taking into account the growth and ongoing development of the child.

With regard to the social system, waiting times for approval of applications for a disabled person’s pass were described as problematic by several interviewees, leading to a delay in entitlement to support benefits.

In the field of education, parents critiqued waiting times for approval of applications for school assistants, whereas children pointed to a lack of continuity of care in this field. They reported frequent changes in staff, stating that the assistant had already changed “*quite often*” (P5 + C3 § 100) or “*always changes*” (C9, § 63). Parents rooted the difficulties with long waiting times and frequent changes of school assistants in a lack of qualified staff and adequate pay. As one parent put it: *“The problem is*,* […] that the school assistants are very poorly paid*,* very poorly qualified*,* and you are only ever lucky if you have someone good”* (P15 § 101). Another major concern was that children were excluded from school when assistants were unavailable, for example due to sick leave or resignation, sometimes for several weeks or even months.

In summary, the accounts of both parents and children highlighted a lack of resources, prevalent in all systems, which prevented children from receiving adequate and timely care.

### Resulting burdens for children and their families

**Fig. 1:**
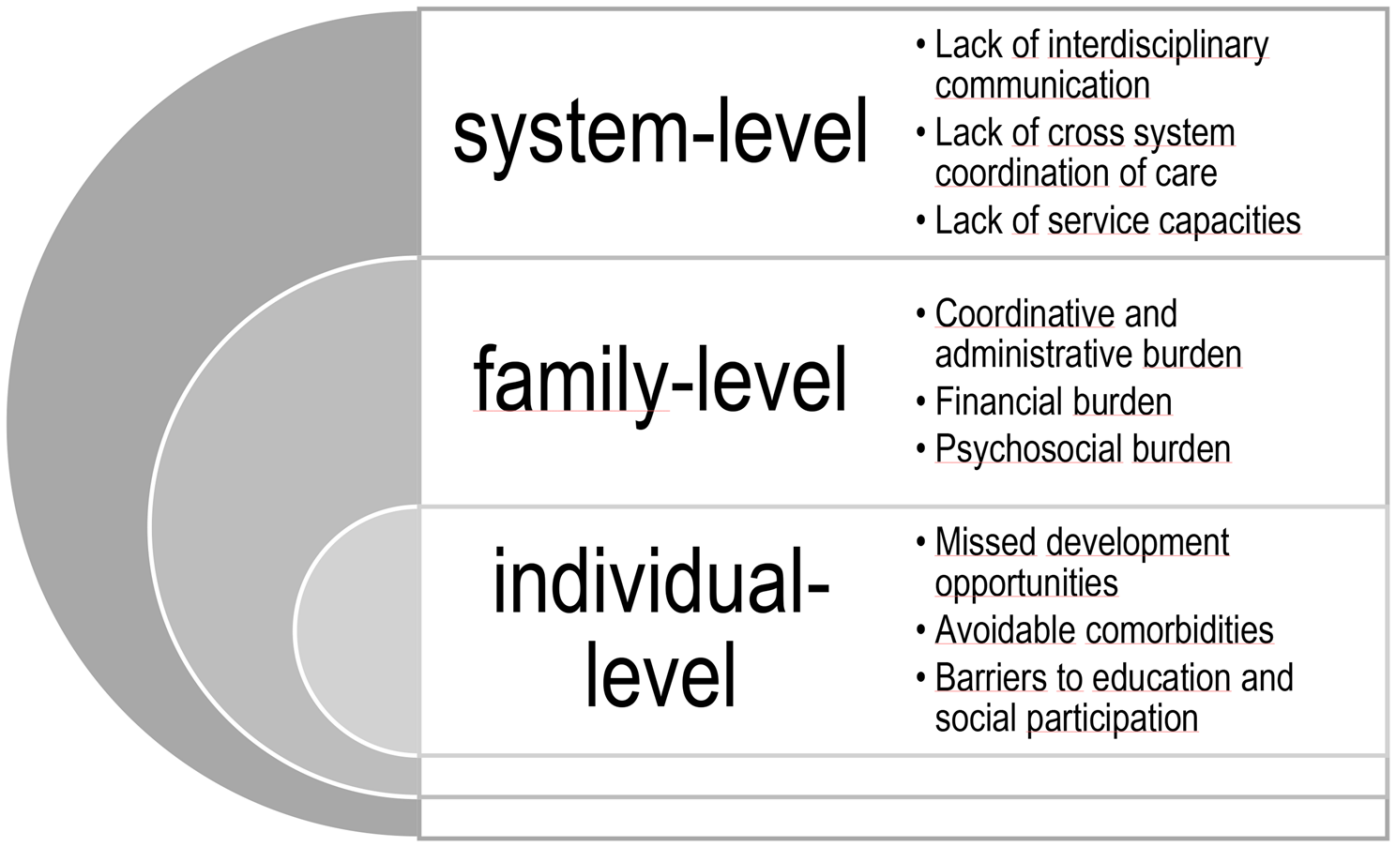
Systemic, familial and individual dimensions of burden in the care of children with chronic conditions

The following section outlines the multidimensional burdens experienced by families as a result of systemic shortcomings, including administrative and logistical demands, financial strain related to balancing employment and caregiving, psychosocial and health-related stress, and restricted access to education and social participation for children. Figure 1 presents an overview over systemic, familial and individual dimensions of burden for families.

### Administrative and logistical burden

The above-mentioned shortcomings in the coordination and organisation of care across system boundaries led to an enormous administrative burden for caregivers. A caregiver of a child with a development disorder said the time spent on administrative effort amounted to “*one day per week*” (P8, § 35). The distribution of benefits and entitlements across the health care, social and educational system made it difficult for caregivers to figure out which benefits the family was entitled to and how they could apply. Parents said they regularly spent hours searching the internet, contacting insurance companies, medical aid providers, authorities and self-help groups until finding the right contact person. Several participants reported not having applied for certain benefits simply because they had no knowledge of it or feared the bureaucratic effort associated with it. Moreover, caregivers felt stalled by authorities and insurance companies through extensive paperwork and long waiting times which made them feel like “*one has to beg and fight for everything*” (P17 § 121). Across all interviews, a central request was the reduction of coordination and bureaucratic burdens for caregivers, for instance through the appointment of a case worker responsible for care coordination, facilitation of interdisciplinary communication among providers, and assistance with benefit applications.

### Financial burden: reconciling paid employment and care responsibilities

The lack of relief measures and support services available on short notice made it challenging for caregivers to pursue paid employment alongside their care responsibilities. Even when their child was in third-party care, e.g., kindergarten or school, caregivers had to remain available to manage their child’s condition, as even special needs schools in Germany do not regularly provide nurses or other staff with medical training. In cases such as clogged insulin pumps or emotional meltdowns, schools often called parents, forcing them to interrupt their work and attend to their child. For children with complex care needs, parents reported they could not find any type of day care and children with behavioural disorders were sometimes even “*excluded [from school] with an administrative order*” (P4 + C2 § 234) – despite compulsory education requirements. These factors combined with the above-mentioned administrative burden made it difficult for parents to find and keep employment. All families included in this study reported that the mother of the child had (at least temporarily) reduced her working hours, taken up self-employment or even given up paid employment entirely, while -with one exception- all fathers were working full-time. Structural conditions were perceived by mothers as contributing to a reinforcement of gendered caregiving norms, as they put primary responsibility for coordinating care and support on families: *“Because that’s always the thing*,* in the end it’s usually the mother and the child*,* isn’t it?”* (P18, § 177). This made mothers financially dependent on their partners and led to a reduction of household income for families.

### Health and psychosocial burden

The burden of care coordination strongly influenced the health and wellbeing of both children and their families. Parents expressed that they felt the responsibility for their child receiving good and adequate care rested on them alone – leading to feelings of stress and exhaustion. Even more so, they felt that there was no support available for them and their child if needed. “*Our experience is that there’s no one there to pick up the slack when we collapse. We simply can’t afford to drop out.”* (P3, § 97). This was a source of worry for many parents of children with complex conditions who reported that the burden associated with organising and providing care for their child took a toll on their personal health. Several parents mentioned suffering from chronic conditions such as chronic back pain, fibromyalgia or anxiety disorders, which were aggravated by sleepless nights due to nocturnal asthma attacks, hypoglycaemia alarms of glucose sensors in diabetic children or circadian dysrhythmia in children with developmental disabilities.

Some children said they suffered from a psychosocial burden associated with their condition. Children with somatic conditions expressed fearing life-threatening emergency situations such as breathlessness, hypoglycaemia or seizures. Others were troubled by rejection from other children, bullying in school or conflicts within the family. Some said they were not able to keep up with peers due to their condition. Across indications, children emphasized they did not want to stick out in front of their peers and did therefore try to avoid tasks related to managing their condition in sight of others, e.g., use of asthma spray.

Caregivers demanded the provision of preventive psychosocial services offered to the whole family as a key component to needs- and prevention-oriented healthcare.

### Barriers to education and social participation

Education and social participation proved to be a major concern for families. Parents described the integration of their child into the school system as difficult. Beginning with barrier-free accessibility of school buildings, over the adaptation of the school curriculum to the abilities and skills of their child to the staffing of the school. They especially criticised the gap between the schools’ claims to inclusion and their families’ lived experiences. *“There is the system with the blinders. And you either fit in or you have to go somewhere else.”* (P9, § 37). This left them worried for their child’s future opportunities and life chances.

The barriers children faced extended to social participation in general, as parents frequently noted. They criticised a lack of barrier-free accessibility and leisure opportunities suitable for their children, stating that their child was “*too disabled”* (P3 § 55) for the options offered. In many cases, parents stated there were no inclusive institutions, playgrounds or leisure activities available in their area: “*Apart from the kindergartens*,* there is really nothing at all.*” (P8, § 47). This made school or kindergarten one of very few -or in some cases the only- social space for children outside the home environment which limited their options to interact with peers and build friendships.

The restricted access to education and social participation made children feel excluded. As one child phrased it: “*Because I’m not the same as the others. It’s as simple as that.*” (P5 + C3 § 25).

**Fig. 2:**
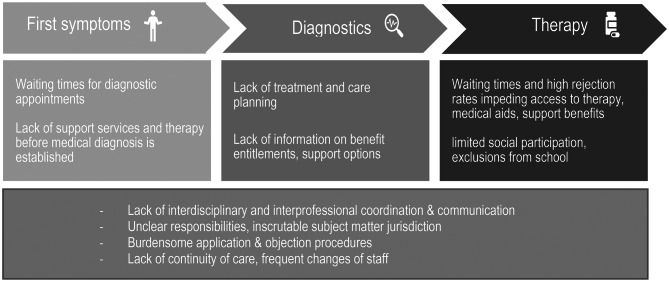
Schematic representation of the patient journey and associated systemic barriers

Children felt that some barriers they faced were rooted in the medical system itself. They reported missing out on a lot of school or leisure activities due to medical appointments. Exemplarily, a child with cerebral palsy reported once attending only two school days in one week because *“the rest of the time I was on the road for the wheelchair*,* handbike*,* all sorts of things”* (C5, § 259). They critiqued healthcare professionals “*talk[ing] past me*” (C5 § 302) during appointments and demanded being included in shared decision-making processes “*because it’s about my life*” (C15 § 177). Figure 2 shows a schematic representation of the patient journey and associated systemic barriers.

## Discussion

This study explored patient journeys of children with chronic conditions from the perspectives of children and their caregivers. Our findings demonstrate how structural conditions within the healthcare, education, and social service system significantly shape the patient journeys of children with chronic conditions. Bureaucratic and administrative barriers -combined with a low degree of digitalisation in Germany- are key factors contributing to caregiver burden, with many families reporting difficulties navigating fragmented systems. In the context of limited paediatric diagnostic and therapeutic capacities, such barriers amplify caregivers’ concerns about missed development opportunities for their children and limited social participation. From the children’s perspective, a lack of inclusive environments—such as barrier-free schools, playgrounds, and leisure activities—reinforce feelings of difference and social isolation. Moreover, frequent medical appointments lead to school absences, contributing to educational disadvantages. These findings underscore the importance of structural reforms aimed at improving system coordination and accessibility to reduce preventable burdens on families and promote equitable care trajectories.

Our results correspond to the existing body of literature on children’s patient journeys in so far as they also highlight the psychosocial burden for children and their families [13, 14, 21, 41]. This is mirrored in studies assessing caregiver burden in parents of children with chronic conditions [3, 4, 42, 43]. A systematic review examining parenting stress in caregivers of children with chronic conditions (e.g. asthma, diabetes, cystic fibrosis) for instance, found greater parental responsibility for treatment management to be associated with an increase of parental stress [3].

In addition to the association with parental health, the study at hand highlights that a reduction of organisational effort for parents is relevant in terms of (health) economic considerations and gendered income differences. The difficulties of reconciling paid employment with care responsibilities outlined here are in line with other studies which found caring for a child with a chronic condition to be leading to parental work absenteeism, reduction in work productivity and negatively impacting caregivers’ health related quality of life [44, 45]. Consistent with the findings in the study at hand, previous studies also found a gender-specific impact on employment patterns between caregivers of children with chronic conditions as compared to caregivers if children without a chronic condition. Mothers of children with different chronic conditions showed a larger reduction of paid working hours than mothers of children without a chronic condition, whereas no difference was observed among fathers [46]. Existing structural gender inequalities in employment seem thus to be aggravated in families with children with chronic conditions [47], pointing to the intersectional nature of the burden experienced by mothers [48], which is insufficiently targeted in existing support services.

Absences from school due to medical appointments which were identified by children in this study as contributing to limitations in social participation, have also been examined in other regional contexts. A study focusing on children with inflammatory bowel disease in the UK found the number of school absences to be significantly higher for affected children than for others – but lower if absences due to scheduled medical appointments were excluded [49]. For the German context, our findings suggest that absence of school assistants may increase absenteeism among children with chronic conditions in addition to absences due to medical appointments, reflecting systemic organisational and structural shortcomings. Hence, to ensure educational participation, a strengthening of services during after-school hours is needed as well as a reliable structure of school assistance services [44]. 

Whereas caregivers emphasized structural barriers to social participation, the accounts of some children illustrated that they tended to internalise their experiences of exclusion, interpreting it as a personal limitation linked to their condition rather than as a consequence of societal inaccessibility and structural hindrances. This internalisation may shape how children understand their experiences, potentially influencing their self-perception and expectations regarding participation.

Long waiting times, complicated procedures and high rejection rates of reimbursement claims which caregivers are compelled to compensate for, put disproportionate burden on families with lower socioeconomic status and thus perpetuate structural inequalities. The substantial resources needed to secure appropriate care and support place these families at a heightened disadvantage regarding access to healthcare, education, psychosocial wellbeing, and their children’s long-term life chances.

The ongoing development of children poses a unique challenge to healthcare systems as a delay in care can result in missed development opportunities. Caregivers and children in this study provided examples of setbacks in therapy and missed opportunities, which make timely care particularly urgent for children.

The comparison across indications showed that child and caregiver burden was not determined by diagnosis, but the severity of the condition and the impact on everyday life of the family. This points to the potential of cross-indication solutions, targeting needs and psychosocial burden not only of the child, but the whole family. While our analysis emphasized cross-indication patterns, some diagnosis-specific differences emerged. Waiting times were reported longer for neuropsychiatric conditions, whereas satisfaction with care coordination was higher among families of children with diabetes, possibly reflecting more standardized care pathways in diabetes care described by families. These nuances, although secondary to the overarching role of severity, highlight potential leverage points for improving care by integrating elements that have proven relevant within the context of specific conditions. An extension of cross-indication, needs-oriented service capacities for children with chronic conditions which are not tied to a specific condition – such as care coordination, psychosocial family support – could help ensure timely, needs-oriented care. Furthermore, future efforts for improving health care for children with chronic conditions should focus on strengthening interdisciplinary and -professional communication as well as care coordination across systems to ensure continuity of care and relieve families of administrative and organisational burden.

Our results highlight unmet needs among children with chronic conditions and their families that perpetuate structural inequalities and call for cross-indication, cross-system solutions. These demands are mirrored in the WHO framework on integrated people-centred health services. Yet, studies on implementation of the WHO framework are scarce [50]. International literature has demonstrated the potential of integrated care approaches to improve quality of care, patient satisfaction, and access to services, particularly for populations with complex care needs [51]. Singular aspects of this comprehensive framework have been implemented in the context of studies on medical homes or complex care models with no coherent results regarding the benefits of the model for children [52–54]. Components of integrated care models examined in studies involving children include needs-based care planning [53, 55, 56], strengthening interprofessional collaboration within and beyond the healthcare sector [57–59], and supporting families with bureaucratic and administrative processes [52, 60]. Our findings suggest that these components should be considered in the development of an integrated care model for children with chronic conditions in the German context. In addition, the barriers to social participation reported by children in our study highlight the importance of extending quality-of-care evaluations for children with chronic conditions beyond the healthcare sector. Specifically, the education system and outcomes such as school absenteeism, as well as physical and systemic barriers to participation, should be incorporated into assessments of care quality and service effectiveness.

While the WHO framework provides a valuable orientation, our results indicate that its implementation in the German healthcare system faces structural challenges. The fragmentation between outpatient and inpatient sectors, divided responsibilities across health and social care systems, and complex financing arrangements may limit the feasibility of seamless care coordination [61, 62]. Implementing more integrated approaches would therefore require not only service-level adjustments but also structural reforms and cross-system governance mechanisms.

### Strengths and limitations

To the best of our knowledge, this is the first study to examine patient journeys of children with chronic conditions from a cross-system, cross-indicational perspective. The qualitative approach and the discussion of results with affected families enabled us to address aspects most relevant for children and their families. In comparison to existing literature on patient journeys which focuses solely on the diagnostic journey, the investigated time period in this study is longer and thus reflects different phases and challenges of children’s journeys. Moreover, most studies focus on a single condition and do not extend beyond the medical sector and thus not capture interface problems at system boundaries or cross-indication characteristics.

However, there are limitations to this study. Despite our efforts to recruit a diverse sample, participants were relatively homogenous in terms of socioeconomic status, migration background, and gender. The response rate was higher for families recruited via social paediatric centres and patient support groups than through other channels. In social paediatric centres, this may reflect the convenience of on-site recruitment, although the interviewer was not employed there or involved in clinical care. In the case of patient support groups, higher participation may be related to the organisations’ advocacy role, with study participation perceived as a way to contribute to the improvement of services for their community. Consequently, the findings may not fully reflect the perspectives and needs of families less well connected to services, for example those experiencing difficulties navigating systems if German is not the family’s first language. Future research should aim to include broader and more diverse samples to better capture the intersectional nature of burden and patient journeys. Nevertheless, our findings provide valuable insights into the structural barriers and resulting burdens for children and their families and may inform the design of a subsequent quantitative study.

## Conclusion

Our findings highlight critical aspects of patient journeys that contribute to child and caregiver burden and to families’ perceptions of declining care quality. Central challenges include fragmented healthcare delivery, poor cross-sector integration, delayed service availability, and a lack of family-centred care. Future research should focus on developing and evaluating concrete models that enable continuous assessment of the needs of children with chronic conditions and their families and that coordinate differentiated support aligned with those needs across medical, educational, and social service systems. The WHO framework on integrated, people-centred health services offers a suitable orientation for such efforts. As cross-system continuity of care seems key, policy efforts should prioritise creating the structural conditions for seamless collaboration across different social and legal systems, including shared terminology, aligned financial and regulatory incentives, and integrated planning and coordination mechanisms.

## Supplementary Information

Below is the link to the electronic supplementary material.

Supplementary Material 1

## Data Availability

The datasets used and/or analysed during the current study are available from the corresponding author on reasonable request.

## Appendix

**Table 1:** Cited participant quotes in original German and English translation

	English Translation	Original German
P5 + C3 § 163	So, somehow a holistic perspective is missing.	Also, es fehlt irgendwie dieser ganzheitliche Blick darauf.
P4 + C2 § 763	It’s like [the game] ‘musical chairs’.	Das ist wie Reise nach Jerusalem
P6 § 73	There are many, many aids, they are just scattered around in a totally incomprehensible system.	Es gibt sehr, sehr viele Hilfen, die sind nur in einem total unübersichtlichen System verstreut.
P3 § 33	The waiting time for a therapy spot is five years, by then L. would almost be an adult.	Die Wartezeit ist bei fünf Jahren auf einen Therapieplatz, dann wäre L. schon fast erwachsen.
P5 + C3 § 100	Yes, quite often.	Ja. Schon häufig.
C9 § 63	Always changes.	Wechselt immer.
P15 § 101	The problem is, […] that the school assistants are very poorly paid, very poorly qualified, and you are only ever lucky if you have someone good	Das Problem ist ja, meines Erachtens, dass die Integrationshilfen sehr schlecht bezahlt werden, sehr schlecht qualifiziert sind, und man immer nur Glück hat, wenn man jemand Gutes hat
C8 § 252	simply because the system doesn’t provide the help at the point where you need it	Und das ist dann weg, einfach weil das System nicht an dem Punkt, wo man die Hilfe braucht, das dann nimmt.
P8 § 35	And I usually have about one day per week that I block for bureaucracy.	Und in der Regel habe ich ungefähr einen Tag in der Woche, den ich mir für Bürokratie freihalte.
P17 § 121	That one has to beg and fight for everything” (P17 § 121).	Dass man um alles betteln und kämpfen muss
P4 + C2 § 234	So in the end, they even excluded her with an administrative order.	Also zum Schluss, sie haben sie sogar ausgeschlossen mit einer Ordnungsverfügung.
P18 § 177	Because that’s always the thing, in the end it’s usually the mother and the child, isn’t it?	Weil das ist immer so, am Ende ist es meistens die Mutter und das Kind, ne?
P8 § 97	Our experience is that there’s no one there to pick up the slack when we collapse. We simply can’t afford to drop out.	Und unsere Erfahrung ist ja auch, es gibt niemanden, der das auffängt, wenn wir ausfallen. Wir dürfen einfach nicht ausfallen.
P9 § 37	There is the system with the blinders. And you either fit in or you have to go somewhere else.	Es gibt das System mit den Scheuklappen. Und entweder passt man da rein oder man hat woanders hinzugehen.
P3 § 55	But L. is too disabled for that, so it didn’t work.	Aber da ist der L. zu behindert für also, es hat nicht funktioniert.
P8 § 47	Apart from the kindergartens, there is really nothing at all.	bis auf die Kindergärten gibt es wirklich gar nichts.
P5 + C3 § 25	Because I’m not the same as the others. It’s as simple as that.	Weil ich nicht genauso bin, wie die anderen. Ganz einfach.
C5 § 259	Last school year / I think I was there two days a week and the rest of the time I was on the road for the wheelchair, handbike, all sorts of things.	Ich war mal letztes Schuljahr / war ich, glaube ich, in der Woche zwei Tage da und den Rest war ich wegen dem Rolli, Handbike, alles Mögliche unterwegs.
C5 § 302	I have more of a problem with the fact that when I’m sitting opposite them and they still talk past me, so to speak.	ich habe eher das Problem damit, dass man, wenn ich ja gegenübersitze und man trotzdem sozusagen an mir vorbeiredet.
C15 § 177	Because it’s about my life.	Weil es halt um mein Leben geht.
