# Supplementary material for: “One has to beg and fight for everything” – a qualitative study on patient journeys of children with chronic conditions in Germany
Source: BMC Health Serv Res. 2026 Mar 20;26:495. doi: 10.1186/s12913-026-14386-5 (PMC13063996; doi:10.1186/s12913-026-14386-5)
Supplement: Supplementary file 1 — Supplementary Material 1 [file 12913_2026_14386_MOESM1_ESM.docx]

**INTERVIEW GUIDE – JOINT PARENT & CHILD INTERVIEW**

**INTRODUCTION - example**

Thank you very much for agreeing to participate in our study.

Addressed to the child: It’s great that you came to talk with us today!

Addressed to both: My name is…. I am part of a team of researchers at the University Hospital Düsseldorf.

Primarily addressed to the parents: In our research project PICAR, we are studying the medical care of chronically ill children between the ages of 3 and 15. In this project, we want to understand what, from your perspective, determines the quality of care and which processes lead to burdens and additional effort or costs. To do this, we conduct interviews with affected children, their parents, and healthcare professionals involved in their care. The results of these interviews will be explored in greater depth in further sub-studies within the project, so that in the end we can develop recommendations for better connected, or “integrated,” care for children with chronic illnesses.

Addressed to the child: So, one could say: Our goal in this project is to improve healthcare. To do that, we would like to find out what you have experienced with your illness when visiting doctors and therapists. You could also say: We are interested in your “journey” through the healthcare system.

Addressed to both: In our conversation today, I would therefore like to talk with you about your experiences on this journey so far and gain an impression of how you have experienced the medical care. Our conversation today will last about one hour and consists of three parts.

Addressed to the parents: First, I will ask you a few questions.

Addressed to the child: After that, I would like to ask you a few things.

Addressed to the parents: And afterward, I would like to ask you a few more questions. If you do not wish to answer a question or would like to end the conversation early, that is possible at any time. Everything we discuss here will remain confidential. No information will be passed on to your doctors, therapists, or health insurance company. With your consent, our interview will be audio-recorded, but without your name. After the interview has been transcribed—that is, written down—the audio recording will be deleted.

Addressed to the child: That means there are no right or wrong answers. We want to know what your opinion is. So that we can analyze it, we will write down the interview and then delete the audio recording. Everything you tell us will always remain within our project team. Only members of our project team can read your interview. No one else—not even your doctors—can read it, and we are not allowed to tell your doctors what you told us.

Addressed to the parents: Do you have any questions about our conversation today?

Addressed to the child: Do you have any questions?

**flexible order: child/parent first**

**PARENTS:**

| **Introduction: Clinical Condition & Diagnosis** | |
| --- | --- |
| **Guiding question** | **Probing questions** |
| Could you briefly describe your child’s medical condition? |  |
| How did it all begin with your child’s illness? | - How much time passed between the first symptoms and the diagnosis? - Which points of contact and professional groups were you in touch with during the diagnostic process? - During the diagnostic process, did you receive a plan outlining how things would proceed for you and your child? |
| **Patient Journey** | |
| **Guiding question** | **Probing questions** |
| How has your journey through the healthcare system developed after the diagnosis? | - Could you elaborate on the timelines involved? - Which points of contact and professional groups were you in touch with? - How did you get to the individual points of contact? How did you find them?   Possible In-Depth Topics   - Who supports you on your journey (private environment / key professional)? - From your perspective, how does communication work between the services involved in care? - Has it ever happened that you did not make use of certain services or did not apply for reimbursement, even though you would have been entitled to them? Why? - Do you feel that the course of your journey has changed in any way due to COVID? |
| **Present and everyday life** | |
| **Guiding question** | **Probing questions** |
| What does your everyday life currently look like? | - Do you have specific routines in your daily life? If so, what are they? - Are there any particular arrangements in school, kindergarten, or childcare due to your child’s illness?   Possible In-Depth Topics   - Do you have domestic help or any other form of support in daily life due to your child’s care needs? Does this result in costs for you? If so, how much, and who covers these costs? - Do you feel that your child is able to participate in all areas of life as he or she would like? - What would your child or your family need in order for your child to participate in all areas of life as you would wish? From your perspective, who would be the appropriate contact person regarding this? |

**CHILD:**

| *Specific situations previously described by the parents can be taken up as reference points in the questions in order to ask the children about their experience of the respective situation and to better contextualize the pre-structured questions for the child.* | |
| --- | --- |
| **Present and everyday life** | |
| **Guiding question** | **Probing questions** |
| I’m interested in what your everyday life looks like. Can you tell me what you usually do on a normal day? | - Are there certain things related to your diagnosis *[if possible, use the word child and parent are using to refer to the condition]* that you have to do every day or every week? - What helps you remember to do those things?   Mögliche Vertiefungsthemen:   - Wie ist das für Dich, mit verschiedenen Ärzt:innen und Therapeut:innen über deine Erkrankung/Diagnose [*möglichst Rückgriff auf von Familie verwendete Terminologie*] zu sprechen? - Wenn Du Termine bei Ärzt:innen oder Therapeut:innen hast, verpasst Du dann manchmal die Schule? - Oder verpasst Du Treffen mit Deinen Freunden? - Falls ja: Wie ist das für Dich? - Sind die Ärzt:innen/Therapeut:innen für Dich gut erreichbar oder brauchst Du lange, um dorthin zu kommen? Wie kommst Du dorthin? Bus/Bahn/Auto/zu Fuß? - Hast Du das Gefühl, es ist wichtig für Dich, zu den Terminen bei Ärzt:innen und Therapeut:innen hinzugehen und dich an deren Empfehlungen zu halten?   Possible Follow-Up Topics  What is it like for you to talk to different doctors or therapists about your condition/diagnosis *[again, use the term the family uses]*?  When you have appointments with doctors or therapists, do you sometimes miss school because of them? Or do you ever miss meetings with your friends?  If yes: What is that like for you?  Are the doctors or therapists easy for you to get to, or does it take a long time to get there? How do you usually get there — by bus, train, car, or walking?  Do you feel that it’s important for you to go to your appointments and to follow the advice the doctors or therapists give you? |

| **Social participation** | |
| --- | --- |
| **Guiding question** | **Probing questions** |

| You mentioned that you go to school/ kindergarten/ childcare. Can you tell me what a usual day is like there? | - Can you take part in everything the way you would like to? - Are you able to do everything you want to do? - If not, why? - How does that feel for you? - What would you need so that you could do everything you want? - What would have to change? - Who could help you with that? |
| --- | --- |

| **Care experiences** | |
| --- | --- |
| **Guiding question** | **Probing questions** |
| Your mom/ dad told me about all the doctors and therapists you’ve seen, and all the times you’ve been at [the doctor’s office, the hospital, the SPZ, etc.]. Can you tell me what it feels like to have all these visits and meet different doctors and therapists? | - Is that okay for you? - Can you tell me when a visit is good or makes you feel comfortable? - Are there times when you feel uncomfortable or things that bother you? - Do you feel like you can decide about your therapy or medicine? - Would you like to be included in making those decisions? |
| If another child had the same condition as you, what do you think shouldn’t happen to them, based on your own experiences? | - Why? |
| Looking ahead, what would you wish for to make things easier or better for you? | - Why? |

**PARENTS:**

| **Care experiences** | |
| --- | --- |
| **Guiding question** | **Probing questions** |
| We are interested in how you have experienced your journey so far, which things you think went well, and what has been difficult for you.  Looking back on your journey so far, how would you evaluate it? | - What has gone well? - Why did you perceive this as positive? - Where would you see the greatest difficulties in your journey? - Why did you perceive this as negative? - In your view, how could these things have been handled better?   Possible Follow-Up Topics   - How have you experienced your participation in decision-making regarding your child’s medical and therapeutic care? How about your child’s participation?   In your view, how well does coordination work between different service providers and payers? Who holds the main responsibility for coordination? Who do you think should have it?   - Do you have an overview of which organization is responsible for covering which costs? How do you manage the reimbursement procedures? What additional information would you like to have? Who should ideally provide this information (e.g., pediatrician, health insurance, etc.)? - Who bears the organizational burden for submitting applications for cost coverage? How extensive is this burden? |
| What would you wish for in the ongoing journey with your child regarding healthcare and services? | - Why? - Is there anything additional that could be offered that would significantly improve your child’s care and reduce your burden? |

| **Parental Burden associated with Child’s Care** | |
| --- | --- |
| **Guiding question** | **Probing questions** |
| When we look at the care of children with chronic illnesses, we know that this also affects parents and families. It often creates additional burdens.  How would you assess your personal burden due to your child’s care? | - How are you personally doing in terms of your own health and well-being? - Can you tell us about the time required to care for your child? What do you use this time for? How is this time spent among the people in your household? - When we talk about costs that may arise from caring for your child, what comes to mind? - What are the biggest cost categories you have personally covered? - How much do you estimate these costs per month? - What percentage of your income do these costs represent? - How does caring for your child affect your work situation? - How many hours per week do you currently work? - Would you work more if you were not responsible for your child’s care? - In the past year, have you received child sickness benefit? - Have you ever called in sick yourself to care for your child? If yes, how often? - Do you receive any support in everyday life due to your child’s care needs? Does this create costs for you? If yes, how much and who covers them?   Possible Follow-Up Topics   - Does the time required for your child’s medical care affect your personal life? - How does your child’s care impact your family situation? |
| **Final Section – Summarizing and Prioritizing** | |

| **Guiding question** | **Probing questions** |
| --- | --- |

| Looking back at everything, from the start of your child’s care to your everyday life today, what would an ideal patient journey look like if there were no restrictions? | - Which points are most important to you? |
| --- | --- |
| Is there anything we didn’t cover today that you feel is important to share? | |

**CHILD:**

| Is there anything we haven’t talked about yet that you would like to tell me? Or anything I should know if I want to find out how a journey like yours could be made better? |
| --- |

**INTERVIEW GUIDE – PARENT INTERVIEW**

Thank you very much for agreeing to take part in our study.

In the PICAR project, we are looking at care pathways, or “patient journeys,” for children with chronic illnesses, aged 3–15 years. Our goal is to understand what, from your perspective, determines the quality of care and which processes create extra effort, burdens, or additional costs. To do this, we conduct interviews with affected children, their parents, and the healthcare professionals involved in their care. The results of these interviews will be explored further in other parts of the project, so that we can eventually develop recommendations for better coordinated, or “integrated,” care for children with chronic illnesses.

We think of medical care pathways as a “journey” — from the moment of diagnosis up to the present day — that you and your child have experienced together. In our conversation today, I would like to talk with you about your experiences on this journey and gain an understanding of how you have experienced your child’s medical care.

Our conversation today will take about one hour. You do not have to answer any question you do not want to, and you may end the interview at any time. Everything we discuss will remain confidential. No information will be shared with your child’s doctors, therapists, or health insurance. With your consent, the interview will be audio-recorded, but without any connection to your name. Once the interview has been transcribed, the audio recording will be deleted.

Do you have any questions before we begin?

| **Introduction: Clinical Condition & Diagnosis** | |
| --- | --- |
| **Guiding question** | **Probing questions** |
| Could you briefly describe your child’s medical condition? |  |
| How did it all begin with your child’s illness? | - How much time passed between the first symptoms and the diagnosis? - Which points of contact and professional groups were you in touch with during the diagnostic process? - During the diagnostic process, did you receive a plan outlining how things would proceed for you and your child? |
| **Patient Journey** | |
| **Guiding question** | **Probing questions** |
| How has your journey through the healthcare system developed after the diagnosis? | - Could you elaborate on the timelines involved? - Which points of contact and professional groups were you in touch with? - How did you get to the individual points of contact? How did you find them?   Possible In-Depth Topics   - Who supports you on your journey (private environment / key professional)? - From your perspective, how does communication work between the services involved in care? - Has it ever happened that you did not make use of certain services or did not apply for reimbursement, even though you would have been entitled to them? Why? - Do you feel that the course of your journey has changed in any way due to COVID? |
| **Present and everyday life** | |
| **Guiding question** | **Probing questions** |
| What does your everyday life currently look like? | - Do you have specific routines in your daily life? If so, what are they? - Are there any particular arrangements in school, kindergarten, or childcare due to your child’s illness?   Possible In-Depth Topics   - Do you have domestic help or any other form of support in daily life due to your child’s care needs? Does this result in costs for you? If so, how much, and who covers these costs? - Do you feel that your child is able to participate in all areas of life as he or she would like? - What would your child or your family need in order for your child to participate in all areas of life as you would wish? From your perspective, who would be the appropriate contact person regarding this? |

| **Care experiences** | |
| --- | --- |
| **Guiding question** | **Probing questions** |
| We are interested in how you have experienced your journey so far, which things you think went well, and what has been difficult for you.  Looking back on your journey so far, how would you evaluate it? | - What has gone well? - Why did you perceive this as positive? - Where would you see the greatest difficulties in your journey? - Why did you perceive this as negative? - In your view, how could these things have been handled better? - Possible Follow-Up Topics - How have you experienced your participation in decision-making regarding your child’s medical and therapeutic care? How about your child’s participation? - In your view, how well does coordination work between different service providers and payers? Who holds the main responsibility for coordination? Who do you think should have it? - Do you have an overview of which organization is responsible for covering which costs? How do you manage the reimbursement procedures? What additional information would you like to have? Who should ideally provide this information (e.g., pediatrician, health insurance, etc.)? - Who bears the organizational burden for submitting applications for cost coverage? How extensive is this burden? |
| What would you wish for in the ongoing journey with your child regarding healthcare and services? | - Why? - Is there anything additional that could be offered that would significantly improve your child’s care and reduce your burden? |

| **Parental Burden associated with Child’s Care** | |
| --- | --- |
| **Guiding question** | **Probing questions** |
| When we look at the care of children with chronic illnesses, we know that this also affects parents and families. It often creates additional burdens.  How would you assess your personal burden due to your child’s care? | - How are you personally doing in terms of your own health and well-being? - Can you tell us about the time required to care for your child? What do you use this time for? How is this time spent among the people in your household? - When we talk about costs that may arise from caring for your child, what comes to mind? - What are the biggest cost categories you have personally covered? - How much do you estimate these costs per month? - What percentage of your income do these costs represent? - How does caring for your child affect your work situation? - How many hours per week do you currently work? - Would you work more if you were not responsible for your child’s care? - In the past year, have you received child sickness benefit? - Have you ever called in sick yourself to care for your child? If yes, how often? - Do you receive any support in everyday life due to your child’s care needs? Does this create costs for you? If yes, how much and who covers them? - Possible Follow-Up Topics - Does the time required for your child’s medical care affect your personal life? - How does your child’s care impact your family situation? |
| **Abschluss – Zusammenfassung und Priorisierung** | |

| **Guiding question** | **Probing questions** |
| --- | --- |

| Looking back at everything, from the start of your child’s care to your everyday life today, what would an ideal patient journey look like if there were no restrictions? | - Which points are most important to you? |
| --- | --- |
| Is there anything we didn’t cover today that you feel is important to share? | |

**INTERVIEW GUIDE – CHILD INTERVIEW**

**INTRODUCTION** (example):

Hi! It’s great that you came to talk with us today!

My name is … and I’m part of a team of researchers at the University Hospital Düsseldorf. We want to help make healthcare better for kids like you. This is part of a “project,” and our project is called PICAR.

In PICAR, we want to find out what your experiences have been like when you went to doctors, therapists, or other healthcare professionals. You could also think of it as your “journey” through the healthcare system. That’s why we want to have a conversation with you today. We call this conversation an interview.

During the interview, we will ask you some questions about your journey through the healthcare system. The most important thing to know is: there are no right or wrong answers. We want to know what you think.

Everything you tell us is private. Only people on our project team can read your interview. Nobody else — not even your doctors — can see it.

To help us understand your answers, we will record the interview. But after we write down everything you said, we will delete the recording. That way, nobody can listen to it anymore.

Do you have any questions before we start?

| **Introduction: Clinical Condition & Diagnosis** | | |
| --- | --- | --- |
| **Guiding question** | **Probing questions** | |
| Can you tell me briefly why you see doctors or get medical care? | - What health condition do you have? | |
| How did the doctors find out that you have this illness?  Possible situations:   - pediatrician’s office - hospital - outpatient care center, e.g., social pediatric center (SPZ) | - Did you notice any signs of your illness yourself? Which ones? - Do you know how long it took from the first time you noticed these things until you got your diagnosis? - When it was found out that you have this illness, how did the doctors or your parents explain what would happen next for you? | |
| **Patient Journey** | | |
| **Guiding question** | **Probing questions** | |
| After you found out about your diagnosis, what was the next step in your care? | - Which doctors or therapists have you been to? - Do you remember how long it took to go from one appointment or place to another? - Was there a doctor or therapist who really helped you or someone you liked going to? - Who has been an important helper or supporter on your journey through medical care? | |
| **Present and everyday life** | | |
| **Guiding question** | **Probing questions** | |
| I’d like to know what a normal day is like for you. Can you tell me what you usually do? | - Are there things you have to do every day or week because of your health? - What helps you remember to do them?   Possible Follow-Up Topics   - Interaction with different doctors and therapists - Scheduling of medical care alongside leisure activities - Accessibility of therapy services - Motivation for therapy | |
| **Social participation** | | |
| **Guiding question** | | **Probing questions** |
| You mentioned that you go to school/ kindergarten/ childcare. Can you tell me what a usual day is like there? | | - Can you take part in everything the way you would like to? - Are you able to do everything you want to do? - If not, why? - How does that feel for you? - What would you need so that you could do everything you want? - What would have to change? - Who could help you with that? |
| **Parental Involvement and Child Autonomy** | | |

| **Guiding question** | **Probing questions** |
| --- | --- |

| Now, you’ve experienced a lot on your journey together with your parents. How was it for you to go through this journey with them? | - Is your condition often something you talk about with your parents? - What is it like for you to go to doctors or therapy appointments with your parents? - Are there appointments that you would rather go to without your parents? - For which appointments is it important to you that your parents are there? |
| --- | --- |

| **Care experiences** | |
| --- | --- |
| **Guiding question** | **Probing questions** |
| Your mom/ dad told me about all the doctors and therapists you’ve seen, and all the times you’ve been at [the doctor’s office, the hospital, the SPZ, etc.]. Can you tell me what it feels like to have all these visits and meet different doctors and therapists? | - Is that okay for you? - Can you tell me when a visit is good or makes you feel comfortable? - Are there times when you feel uncomfortable or things that bother you?   Possible Follow-Up Topics:   - Do you feel like you can decide about your therapy or medicine? - Would you like to be included in making those decisions? |
| If another child had the same condition as you, what do you think shouldn’t happen to them, based on your own experiences? | - Why? |

| Looking ahead, what would you wish for to make things easier or better for you? | - Why? |
| --- | --- |
| Is there anything we haven’t talked about yet that you would like to tell me? Or anything I should know if I want to find out how a journey like yours could be made better? | |
